# Randomized phase III trial of radiotherapy or chemoradiotherapy with topotecan and cisplatin in intermediate-risk cervical cancer patients after radical hysterectomy

**DOI:** 10.1186/s12885-015-1355-1

**Published:** 2015-05-04

**Authors:** Wenze Sun, Tao Wang, Fan Shi, Jiquan Wang, Juan Wang, Beina Hui, Yingbing Zhang, Jinli Lu, Hongwei Chen, Zi Liu

**Affiliations:** Department of Radiation Oncology, First Affiliated Hospital of Xi’an Jiaotong University, 277 Yanta West Road, Yanta District, Xi’an, 710061 China

**Keywords:** Chemoradiotherapy, Topotecan, Cisplatin, Intermediate-risk, Cervical cancer

## Abstract

**Background:**

In cervical cancer patients with intermediate-risk factors, the optimal adjuvant therapy is still controversial. We undertook a randomized trial (ClinicalTrials.gov Identifier: NCT01418859) to compare the efficacy and toxicity of concurrent chemoradiotherapy with topotecan and cisplatin with radiotherapy alone in intermediate-risk cervical cancer patients.

**Methods:**

Eligible patients were randomly assigned to one of three treatment arms including arm A (radiotherapy only,RT), arm B(concurrent chemoradiotherapy only, CCRT), and arm C (concurrent chemoradiotherapy with following consolidation chemotherapy, CCRT + CT). All eligible patients completed external RT (IMRT or 3D-CRT), receiving 45-50Gy /25f uniformly to the pelvis. Concurrent chemotherapy regimen was topotecan 0.75 mg/m^2^ for days 1, 2 and 3, followed by cisplatin 25 mg/m^2^ for days 1, 2 and 3. Three cycles of consolidation chemotherapy regimen was topotecan 1.5 mg/m^2^ for days 1 and 2, and 0.75 mg/m^2^ for day 3; followed by cisplatin 25 mg/m^2^ for days 1, 2 and 3, repeated every 21 days. Adverse events of each group were investigated and compared.

**Results:**

Thirty-nine patients enrolled onto the remaining regimens: 14 to RT, 15 to CCRT and 10 to CCRT + CT. Six patients (15.4%) did not complete the protocol treatment. Hematologic toxicity was more frequent and more severe in the CCRT and CCRT + CT arms compared with the RT arm. The incidence of grade 3-4 neutropenia was significantly different statistically between the RT, CCRT and CCRT + RT groups (15.4%, 46.7% and 100%, respectively; *P* = 0.002). Specially, three patients in CCRT + CT arm of all six patients who did not complete the protocol treatment discontinued planned therapy because of persistent grade 4 neutropenia. However, there were no significant differences in grade 3-4 non-hematologic toxicities between the three groups(all *P* > 0.05). Recurrence-free survival and overall survival of each group were not analyzed on account of a median follow-up of only 16 months.

**Conclusions:**

Concurrent chemoradiotherapy with topotecan and cisplatin showed severe hematologic toxicity in intermediate-risk cervical cancer patients after radical hysterectomy. Thus, the study was closed ahead of schedule.

**Trial registration:**

ClinicalTrials.gov Identifier: NCT01418859.

## Background

Radical hysterectomy with pelvic lymphadenectomy has been widely accepted as treatment in patients with stage IB-IIA cervical cancer. However, the survival of patients with early stage cervical cancer after radical hysterectomy and pelvic lymphadenectomy depends on the presence or absence of several poor prognostic clinicopathologic risk factors. Positive or close resection margin, lymph node involvement, and parametrial invasion were recognized as high-risk factors while large tumor size, deep stromal invasion, and lymphovascular space invasion (LVSI) were regarded as intermediate-risk factors [1].

Radiation has been used as a postoperative adjuvant therapy to reduce recurrences in cervical cancer patients with both these intermediate and high risk factors [2-5]. Since Peters et al. showed a significant survival advantage with the use of concurrent chemoradiation rather than radiation in patients with high-risk cervical cancer [6]. Concurrent chemoradiation rapidly replaced radiation in the treatment of high-risk cervical cancer [6-8].

Although radiotherapy has been used as postoperative adjuvant therapy to reduce recurrence and improve survival in patients with intermediate risk tumors and the effectiveness of radiotherapy has been widely accepted, based on the results of a randomized study reported by Sedlis et al. [9]. Recent retrospective studies insisted that adjuvant radiotherapy may have local effects only, which are not effective in distant recurrence and have suggested the promising improvements of adding chemotherapy to radiation in patients with intermediate-risk factors [10-12]. Thus, two practical debates still remain regarding for the adjuvant treatment of the intermediate-risk group. First, is whether concurrent chemoradiotherapy (CCRT), which has been established as a standard adjuvant treatment of high-risk cervical cancer, is more beneficial than radiotherapy only for an intermediate-risk group. Second, cisplatin-based chemotherapy usually has been accepted as effective adjuvant therapy against cervical cancer. However, the optimal platinum-based combination treatments in adjuvant concurrent chemoradiation (CCRT) is still under investigation. Topotecan is an agent with antineoplastic activity on recurrent or metastatic cervical cancer and has been shown acceptable toxicity and good response rates in previous studies as a combined regimen with cisplatin [13,14]. Consequently, topotecan/cisplatin was chosen as concurrent chemotherapy regimen in our study.

The aim of the present study (ClinicalTrials.gov Identifier: NCT01418859) was to investigate the efficacy and safety of CCRT with topotecan/cisplatin as adjuvant therapy after radical hysterectomy for cervical cancer patients with intermediate-risk factors.

## Methods

### Eligibility

Patients from First Affiliated Hospital of Xi’an Jiaotong University who had undergone radical surgery for clinical stage IB- IIA carcinoma of the cervix were enrolled in the study from September 2011 to August 2013. Radical hysterectomy and pelvic lymphadenectomy, with or without para-aortic lymphadenectomy was performed either via laparotomy or laparoscopy, according to the Gynecologic Procedures Manual of the GOG, which outlined minimum surgical requirements for study entry [15]. Patients who had biopsy-confirmed or were strongly suspected of para-aortic node metastasis on preoperative CT or MRI scan were not eligible. A total of 39 patients who had squamous carcinoma, adenocarcinoma, or adenosquamous carcinoma were eligible and all had histologically confirmed the following intermediate risk factors: greater than one-third stromal invasion, lymphovascular space involvement, or tumor size ≥4 cm. Patients who had high-risk factors, such as positive pelvic lymph nodes, parametrial involvement, or positive surgical margins, were excluded. Registration was required within 4-6 weeks of surgery, with RT to begin within 3-5 working days of registration.

Patients were required to have an Eastern Cooperative Oncology Group performance status of 0 to 2. The research was approved by Institutional Ethics Committee of First Affiliated Hospital of Xi’an Jiaotong University. The written informed consent was obtained from each patient.

### Treatment

Following registration, patients were randomly assigned to one of three treatment arms including arm A (radiotherapy only), arm B (concurrent chemoradiotherapy only), and arm C (concurrent chemoradiotherapy with following consolidation chemotherapy).

All the patients underwent external beam radiation therapy to whole pelvis, but no brachytherapy. The external RT was given using IMRT or 3D-CRT with x-ray energy of 10 MV and consisted of 180-200 cGy per day on days 1 to 5 of each week, for a total of 25 fractions. Radiation dose ranged from 4500 to 5000 cGy. Radiation therapy was withheld if a patient had an absolute neutrophil count < 500/mm^3^, and delays of up to 5 days were also allowed in the event of radiation-related gastrointestinal or genitourinary toxicity.

One cycle of chemotherapy was administered at the second week concurrent with RT as follows:topotecan 0.75 mg/m^2^ intravenously during 30 minutes, followed by cisplatin 25 mg/m^2^ intravenously for days 1, 2 and 3.

Three cycles of consolidation chemotherapy were performed followed by first concurrent chemotherapy at the 6th, 10th and 14th weeks. The regimen was topotecan 1.5 mg/m^2^ for days 1and 2, and 0.75 mg/m^2^ for day 3; followed by cisplatin 25 mg/m^2^ for day 1, 2 and 3, repeated every 21 days.

The protocol is registered with ClinicalTrials.gov, number NCT01418859.

### Treatment modifications

Dose adjustment was based on the greatest toxicity grade, using the National Cancer Institute Common Toxicity Criteria for Adverse Events (version 3). Chemotherapy was performed, providing the patient’s absolute neutrophil count recovered to ≥1500/mm^3^ and platelets were ≥100,000/mm^3^ on the day of re-treatment. The cisplatin dose was decreased by 50% for grade 2 renal toxicity and held for the present cycle for grade 3 to 4 renal toxicity on the scheduled day of re-treatment. While topotecan was reduced by 20% for grade 3 and by 40% for grade 4 interval hematologic toxicity for the entire course of therapy. No dose reductions were allowed for grade 1 or 2 interval hematologic toxicity. Chemotherapy was also withheld until resolution of any grade 3 or 4 nonhematologic toxicity.

### Analysis

Between the radiotherapy, chemoradiotherapy, chemoradiotherapy plus consolidation chemotherapy groups, the differences of clinicopathologic variables were evaluated using the chi-square test, Fisher’s exact test, or linear by linear association method for categorical variables. Adverse event rates of the three groups were compared using Fisher’s exact test. All analyses were performed using SPSS 17.0 software (SPSS Inc., Chicago, IL) and *P* values less than 0.05 were considered significant.

## Results

### Treatment and compliance

Of the remaining 39 eligible patients (stage IB-IIA), 14 were randomly allocated to receive radiotherapy only (arm A), 15 were randomly allocated to receive chemoradiotherapy (arm B) and 10 were randomly allocated to receive chemoradiotherapy plus consolidation chemotherapy (arm C). Among patients randomly allocated to receive radiotherapy, chemoradiotherapy or chemoradiotherapy plus consolidation chemotherapy, patient characteristics were well-balanced (Table 1).

**Table 1:** **Patient characteristics**

Clinicopathologic variables	RT	CCRT	CCRT + CT	*P* value
	(N = 13)	(N = 15)	(N = 5)	
**Age**				
≤50	3	4	1	0.558
>50	10	11	4	
**Cell type**				
Squamous	12	13	5	0.329
Non-squamous	1	2	0	
**Tumor grade**				
I	1	1	0	0.583
II	9	10	3	
III	3	4	2	
**Stage**				
IB1	1	2	0	0.782
IB2	8	9	3	
IIA1	2	3	1	
IIA2	2	1	1	
**Risk factors**				
LTS only	1	2	0	0.489
DSI only	7	7	3	
LVSI only	0	0	0	
LTS + DSI	5	5	2	
LTS + LVSI	0	0	0	
DSI + LVSI	0	1	0	
LTS + DSI + LVSI	0	0	0	

*Abbreviations:RT* radiotherapy, *CCRT* chemoradiotherapy, *CCRT + CT* chemoradiotherapy plus consolidation chemotherapy, *LTS* large tumor size, *DSI* deep stromal invasion, *LVSI* lymphovascular space involvement.

Fifteen percent of patients (6 of 39) did not complete protocol therapy. Three patients discontinued planned therapy in arm C because of persistent grade 4 neutropenia toxicity. Other three patients also did not complete the therapy, including one in arm A because of costs of treatment and two in arm C because of refusal to continue. Therefore, 33 patients, 13 in arm A, 15 in arm B, and 5 in arm C completed treatment protocol and were analysed.

Radiotherapy was withheld in 12 patients owing to grade 4 hematologic toxicity or for 5 to 9 days until neutropenia recovered to grade 2. Chemotherapy was suspended because of grade 4 hematologic toxicity in 7 patients during consolidation chemotherapy. Specially, three patients discontinued planned therapy in arm C because of persistent grade 4 neutropenia.

Recurrence-free survival and overall survival of each group were not analyzed on account of a median follow-up of only 16 months.

### Toxicity

Adverse events are summarized in Table 2. Hematologic toxicity was more frequent and more severe in the CCRT and CCRT + CT arms compared with the RT arm. Grade 3 and 4 neutropenia occurred in 46.7% (7/15) of patients who received CCRT and 100.0% (5/5) of patients who received CCRT + CT but in only 15.4% (2/13) of those who received RT only (*P* = 0.002). Febrile neutropenia occurred in 40.0% (2/5) of patients treated with CCRT + CT and 14.3% (1/7) of those who received CCRT. Grade 3 and 4 thrombocytopenia occurred in 26.7% (4/15) of patients who received CCRT and 40.0% (2/5) of patients who received CCRT + CT versus only 7.7% (1/13) of those who received RT. (*P* = 0.079). Dose reductions were required for all the 5 patients in CCRT + CT group in a subsequent cycle. However, there were no significant differences in grade 3 or 4 non-hematologic toxicities between the three groups. Diarrhea grades 3 and 4 were observed in1 (7.6%) patients of RT group, in 3 (20.0%) patients of CCRT group and in 1 (20.0%) patient of CCRT + CT group, respectively (*P* = 0.383). Genitourinary grades 3 and 4 occurred in only 1 patient in each arms (*P* = 0.673).

**Table 2:** **Adverse events by treatment**

Adverse event	RT		CCRT		CCRT + CT		*P* value
	(Grade),N = 13		(Grade),N = 15		(Grade),N = 5		
	3	4	3	4	3	4	
Neutropenia	2	0	2	5	3	2	0.002
Anemia	1	0	2	0	1	0	0.128
Thrombocytopenia	1	0	3	1	1	1	0.079
Nausea/vomiting	1	0	2	0	1	0	0.446
Diarrhea	1	0	2	1	1	0	0.383
Genitourinary	1	0	1	0	1	0	0.673
Neurotoxicity	0	0	0	0	0	0	1
Renal	0	0	0	0	0	0	1
Hepatic	0	0	1	0	1	0	0.339

*Abbreviations:RT* radiotherapy, *CCRT* chemoradiotherapy, *CCRT + CT* chemoradiotherapy plus consolidation chemotherapy.

## Discussion

Radical hysterectomy with pelvic lymphadenectomy has been widely accepted as the treatment in patients with IB-IIA cervical cancer. Because 10-20% of the patients recurred after radical surgery, efforts to reduce recurrences and improve survival in patients with cervical cancer have been made [11].

Since the beneficial effect of adjuvant concurrent chemoradiation for cervical patients with high-risk factors was proven in prospective studies from the Gynecologic Oncology Group (GOG) and the Radiation Therapy Oncology Group [3,6], concurrent chemoradiation is considered the optimum postoperative adjuvant therapy in patients with high-risk factors. However, in a Gynecologic Oncology Group study(GOG 92) that attempted to investigate the role of adjuvant radiotherapy (RT) in the intermediate-risk group, 277 cervical cancer patients with intermediate risk factors were randomized to the no further treatment (NFT) or RT group [9]. In those who met the GOG criteria, which defined the intermediate-risk group using a three-factor model of a combination of intermediate risk factors (capillary lymphatic space involvement, stromal invasion depth, and tumor size), a statistically significant reduction of risk of recurrence and an increase in recurrence free survival was found in the RT group compared with the NFT group. Since the efficacy of adjuvant radiation for patients with intermediate-risk factors were demonstrated in the previous trials [5,9,11,12], radiation is the standard adjuvant therapy in patients with intermediate-risk factors. However, as shown in the GOG 92 trial, the recurrence rate of the intermediate-risk group was 27.9% in the NFT group, comparable to that of patients with high-risk factors. Furthermore, even though the recurrence rate was reduced with the use of adjuvant RT, the rate of recurrence was still significant (15%, 21 of 137 cases) in the RT group [11]. In addition, in the study of Tuipae S et al., distant recurrence was still the major pattern of treatment failure after adjuvant radiotherapy in cervical cancer with intermediate risk factors [12].

Maybe the adjuvant chemoradiation have more beneficial effect over radiation only for patients with intermediate risk factors. An early study reported that, in patients with LVSI and deep stromal invasion, the 3-year RFS (recurrence-free survival) of the concurrent chemoradiation group was significantly greater than that of the radiation group [16]. Some retrospective studies in recent years also have showed the benefit of adding chemotherapy to radiation in patients with intermediate-risk factors and the levels of all toxicities were acceptable [17-20]. A statistically significant difference was found in the 3-year RFS rate among the no further treatment, RT, and CCRT groups (67.5%, 90.5%, and 97.5%, respectively; *p* <0 .05) in the study of Ryu SY et al. [18]. Okazawa M et al. reviewed the medical records of 316 patients with stage IB1-IIB cervical cancer who had been treated with adjuvant radiotherapy (RT) (n = 124, RT group) or adjuvant CCRT (n = 192, CCRT group) after radical hysterectomy and found that CCRT was superior to RT with regard to recurrence rate and PFS in patents with 2 or more intermediate risk factors, while the patients with only 1 intermediate risk factor showed no survival benefit of CCRT over RT [20]. However, due to the lack of randomized trials, the benefit of concurrent chemoradiation over radiation in cervical cancer patients with intermediate-risk factors remains unclear.

Furthermore, adjuvant CCRT modalities for intermediate risk tumors have been debating. Weekly cisplatin or cisplatin plus 5-fluorouracil (5- FU) every 3 weeks has been widely used as the preferred treatment with CCRT in previous studies. Nevertheless, these standard regimens had some disadvantages [21,22]. For instance, the weekly cisplatin frequently caused a treatment delay due to hematologic toxicities and the requirement of a weekly visit to the hospital was inconvenient. In addition, 5-fluorouracil/cisplatin, a five-day regimen, caused more discomfort to patients and sometimes caused intractable nausea and vomiting. While previous studies using topotecan/cisplatin for patients with recurrent or metastatic cervical cancer have shown acceptable toxicity and good response rates. Thus, topotecan with cisplatin was chosen as concurrent chemotherapy regimen in our study.

Recently, cisplatin-based consolidation chemotherapy after CCRT has been reported to enhance local control and promote eradication of possible distant micro-metastases in locally advanced cervical cancer by phase II studies [23-25]. These promsing results suggested that consolidation chemotherapy after primary CCRT may play a role independent of the radiotherapy in locally advanced cervical carcinoma. Furthermore, for high-risk patients after radical surgery, the continuation of chemotherapy (cisplatin 70 mg/m^2^ on day 1 and 5-FU 1000 mg/m^2^/d for 5 days) for three to four cycles resulted in improved patient survival compared with the survival of patients who receive only one or two courses of chemotherapy(*P* < 0.03) in the study of Peters 3rd WA et al. [3]. It was not determined from their study whether the effect of the chemotherapy was as a radiation sensitiser or as a systemic chemotherapy with elimination of possible micrometastasis, or both. In contrast, Kim HS et al. found that consolidation chemotherapy after CCRT using paclitaxel and carboplatin failed to improve PFS and OS as compared to CCRT-only in patients with high-risk early-stage cervical cancer who underwent surgical treatment (3-year PFS, 62.7% vs. 88.2%, respectively; 3-year OS, 90.9% vs. 97.3%, respectively) [26]. However, is there any role for consolidation chemotherapy following CCRT in intermediate-risk cervical cancer patients? We designed arm C(CCRT + CT) to examine whether the addition of consolidation chemotherapy to CCRT could improve the progression-free survival and overall survival in patients with intermediate-risk factors after radical surgery.

On account of a median follow-up of only 16 months, RFS (recurrence-free survival) and OS (overall survival) of each group were not analyzed in the present study. Another issue that must be discussed is complications of these adjuvant therapies following radical surgery. Although cisplatin-based chemotherapy concurrent with RT is a standard treatment modality for advanced-stage cervical cancer, many physicians are still reluctant to treat the intermediate-risk group with CCRT. Because combined modalities are more likely to result in serious complications. As is known, radiotherapy after radical hysterectomy has been reported to be associated with high incidence of complications and many of these conditions are very tragic and irreversible and result in poor quality of life [27,28]. Barter et al. observed that 30% of patients treated with radiation after radical hysterectomy had serious complications, such as gastrointestinal and genitourinary complications and leg lymphedema et al. [28]. Meanwhile, the major toxicities of combined chemotherapy were bone marrow depression, mild to moderate gastrointestinal troubles, mild to moderate nephrotoxicities, total alopecia (in etoposide-cisplatin group), relatively low incidence of neurotoxicities and hepatotoxicities, rare incidence of pulmonary and cardiac toxicities and some reported secondary malignancies (acute leukemia and some solid tumors). In previous studies the levels of all these toxicities of combined chemotherapy were acceptable and most are reversible and transient [17-20]. Nevertheless, the results of the present study found that hematologic toxicity was significantly more frequent and more severe in the CCRT and CCRT + CT arms compared with the RT arm. Grade 3 and 4 neutropenia occurred in 46.7% (7/15) of patients who received CCRT and 100.0% (5/5) of patients who received CCRT + CT but in only 15.4% (2/13) of those who received RT only (*P* = 0.002). Especially, three patients discontinued planned therapy in arm C because of persistent grade 4 neutropenia toxicity. Although topotecan with cisplatin has been shown acceptable toxicity and good response rates in recurrent or metastatic cervical cancer, similar to the study of Long HJ 3rd et al., which reported that the combination of topotecan-cisplatin resulted in substantial neutropenia (70% *vs* 1.4%) compared with single cisplatin agent, and nearly 18% of patients in the topotecan-cisplatin arm experienced febrile neutropenia in patients with advanced (stage IVB), recurrent or persistent carcinoma of the uterine cervix, topotecan plus cisplatin chemotherapy concurrent with RT in our study also showed severe hematologic toxicity [14]. Therefore, we closed the clinical trial ahead of schedule.

Although our study showed severe hematologic toxicity of concurrent chemoradiation with topotecan and cisplatin in safe dose to radiation in patients with intermediate-risk factors, the efficacy and toxicity are still unclear. As we mentioned in the results, the number of patients in the different group is small (14 in group A, 15 in group B and 10 in group C) because the study closed ahead of schedule and the media follow-up time is only 16 months. In spite of these limitations, due to topotecan has been identificated as an antineoplastic agent with cisplatin in cervival cancer, a trial with optimal dose, sufficient size and long term follow-up would be necessary to demonstrate the advantage of concurrent chemoradiation in patients with intermediate-risk factors.

## Conclusions

To conclude that chemoradiotherapy maybe the better adjuvant therapy than radiation in patients with intermediate-risk factors, but the benefit of chemoradiotherapy should overweigh the cost of adverse effects. The present study showed severe hematologic toxicity in the chemoradiotherapy with topotecan and cisplatin groups and was closed ahead of schedule. Therefore, the optimal platinum-based adjuvant chemoradiotherapy in cervical cancer patients with intermediate risk factors is still under investigation especially and the final conclusion regarding the role of chemoradiotherapy should be withheld.

## Abbreviations

RT: Radiotherapy
CCRT: Concurrent chemoradiotherapy
CCRT + CT: Concurrent chemoradiotherapy with following Consolidation chemotherapy
IMRT: Intensity modulated radiation therapy
3D-CRT: 3-dimensional conformal radiotherapy
LTS: Large tumor size
DSI: Deep stromal invasion
LVSI: Lymphovascular space invasion
GOG: Gynecologic Oncology Group
CT: Computed tomography
MRI: Magnetic resonance imaging
NFT: No further treatment
RFS: Recurrence-free survival
OS: Overall survival
